# T-scans in implant procedures

**DOI:** 10.6026/97320630019035

**Published:** 2023-01-31

**Authors:** Prabhav Kumar Iyer, Suresh Venugopalan, Thiyaneswaran N, Sam Jebaraj

**Affiliations:** 1 Saveetha Dental College, Saveetha Institute Of Medical and Technical Science, Saveetha University, 162, Poonamallee High Road, Chennai, India

**Keywords:** implant, occlusion, T scan, contact, innovation

## Abstract

T scans are a breakthrough in technology which allows the user to accurately analyze the occlusion of a patient with minimal to zero errors. They are used during prosthodontic rehabilitation to map out the patient's occlusion to plan for replacements. Such a high advancement in technology has a backdraw. The cost of the equipment is a concern for simple dental practitioners. There are no large scale studies using T scans in determining the occlusion. Therefore, it is of interest to analyze the use of T scans in determining the percentage of contact in patients with dental implants. This retrospective analysis was done at the Saveetha University hospital set up where patients undergoing prosthesis fabrication for implants were included. Details like their age, gender and the percentage of contact of the implant and percentage of contact on the adjacent tooth were recorded. These details were tabulated and imported to IBM SPSS version 23 for statistical analysis. Chi square test was used to analyze comparable variables. 22 patients were analyzed, the mean percentage of contact of implants was found to be 7.45±;9.01 and the mean percentage of contact of adjacent teeth was found to be 10.14±6.7. Thus, T scan is an efficient method in recording the dynamic occlusal contacts of a patient. The use of T scan reveals data pertaining to individual tooth but in the present study there is no statistical significance in terms of exact values comparing implant contact to adjacent tooth contact. Further studies are needed with relation to timing and contact surface of implant prosthesis.

## Background:

The survival rate of the dental implants is correlated with the surgical technique, the osseo integration and the correct execution of the prosthetic restoration [1, 2]. The occlusion of this restoration must be done properly, so the forces that appear do not cause the breakdown of the osseo integration [3]. It has been recommended that the occlusal morphology should have a smooth shape with minimal cusp height and fossae depth [4]. Knowledge about the pattern of tooth contact, properties of material and methods used to record said tooth contact are necessary for accurate examination of occlusion in prosthodontic treatment [5]. The argument of solely relying on T scans to record occlusal forces distribution for implant planning is a debate as the costs of a T scan exceeds the budget of most simple dental practices. However, a few studies have shown that the standard method produces a lot of errors [6]. The T-scan system utilizes a 0.1-mm-thick sensor made with a flexible material that can prevent errors of mandibular deviation caused by excessively thick or hard sensors when occluding [7]. The parameters of the T-scan III system, including introduction time, occlusal center, track of occlusal center, and percentage of occlusal force distribution, can provide a reference for analyzing instant occlusal conditions [7]. These parameters can also form a time track distribution of the points of maximum occlusion and occlusal force through contact of the teeth with the sensor [7]. This information is beneficial for quantitative research in occlusal equilibrium. The T-scan III system sensor comprises two polyester films as substrate, conductive ink as an interlayer, and a vertical and horizontal woven wire grid with approximately 1500 sensor points [7]. Since polyester films have high tear and strain resistance, the thin polyester films in the T-scan III system can endure occlusal force and change form during occlusal movement [7]. The sensor is approximately 60µm thick, which does not hinder the subjects when conducting different types of occlusal exercises [7]. When occlusal force is applied to the sensor, the sensor points change local electronic resistance due to stress. The system measures the changes in current loops [7]. After collecting the data, corresponding software can be utilized to conduct quantitative analysis of the changes in occlusal contact points and force overtime and then compute a distribution of the occlusal forces [7]. However, there are not enough large scale studies which look into the use of T scans in patients with dental implants as, as discussed above, the cost that the practitioner has to bear to buy and maintain a T scan unit and the cost that a patient has to bear to get a scan taken is usually over the price limit of both, the practitioner and patient. Hence, the aim of this study is to analyze the use of T scans in determining the percentage of contact in patients with dental implants.

## Materials and Methods:

The present study was done in the form of a retrospective analysis in a university dental hospital where patients undergoing prosthesis for dental implants were included. Patients who had crowns placed for implants and for whom T scans were taken were included in the study. Details like their age, gender and the percentage of contact of the implant and percentage of contact on the adjacent tooth were recorded. These details were checked by an internal reviewer to remove any bias. The recorded details were tabulated and imported to IBM SPSS version 23 for statistical analysis. Chi square test was used to analyze comparable variables.

## Results:

Our team has extensive knowledge and research experience that has translate into high quality publications [8, 9, 10 11, 12, 13, 14, 15, 16] [17, 18, 19, 20, 21, 22 [23, 24, 25, 26, 27]. The dentists are very interested in the correlation between the patient's habitual occlusion and the related biomechanical elements of it [28]. Some authors claimed that the attempts to measure bite forces is an inaccurate science and that often the dentists have to make and assume complicated decisions based on such subjective appreciation [29]. The unequal distribution of the occlusal forces on teeth that are not making simultaneous contacts is resulting in occlusal trauma. It can appear either on the intact teeth because of the incorrect occlusal contacts, or on the incorrect over contoured restorations that determine a bite raising [30]. The study was con ducted analyzing the data of 22 patients with dental implants who had a T scan taken to check for occlusion during fabrication of prosthesis for the implants. In this study, the mean percentage of contact of implants was found to be 7.45±9.01 and the mean percentage of contact of adjacent teeth was found to be 10.14±;6.7. A study by et al Montero et al showed similar results when mean POC was measured [31]. But, a similar study done by Sequeros et al. showed that no premature contact existed in patients with dental implants [29]. In this study, there was no relation between the POCs and age and gender and no association was found between POC of implant and POC of adjacent tooth to implant; no difference was found which was statistically significant. There is a lack of studies which focus on the percentage of contact of implants and that on adjacent teeth measured using T scans. The limitation of the current study is the small sample size and its participants belonging from similar geographic locations. Future studies done overcoming these limitations might yield different results.

## Conclusion:

T scan is an efficient method in recording the dynamic occlusal contacts of a patient. The use of T scan reveals data pertaining to individual teeth. However, results in this study show no statistical significance in terms of exact values compared to implant contact to adjacent tooth contact. Further studies are needed with relation to timing and contact surface of implant prosthesis.

## Figures and Tables

**Figure 1:**
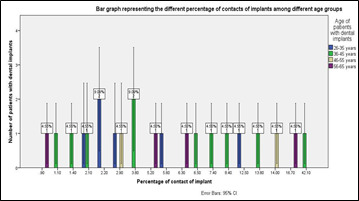
Bar graph representing the different percentages of contact of implants among different age groups. The X axis represents the various percentage of contact of implants and the Y axis represents the number of patients with dental implants. Blue represents patients between the ages of 22-35 years, green represents patients between the ages of 36-45 years, beige represents patients between the age of 46-55 years and violet represents patients between the ages of 56-65 years. p value was found to be 0.30 (p>0.05) which is not statistically significant.

**Figure 2:**
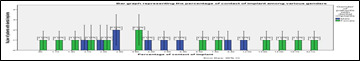
Bar graph representing the different percentages of contact of implants among different genders. The X axis represents the various percentage of contact of implants and the Y axis represents the number of patients with dental implants. Blue represents the male patients and green represents the female patients. p value was found to be 0.39 (p>0.05) which is not statistically significant.

**Figure 3:**
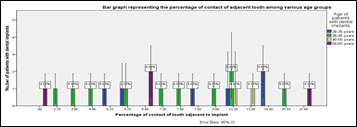
Bar graph representing the different percentages of contact of adjacent tooth among different age groups. The X axis represents the various percentage of contact of implants and the Y axis represents the number of patients with dental implants. Blue represents patients between the ages of 22-35 years, green represents patients between the age of 36-45 years, beige represents patients between the age of 46-55 years and violet represents patients between the age of 56-65 years. p value was found to be 0.28 (p>0.05) which is not statistically significant.

**Figure 4:**
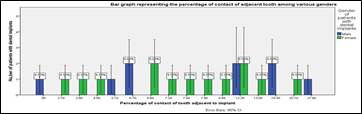
Bar graph representing the different percentages of contact of adjacent tooth among different genders. The X axis represents the various percentage of contact of implants and the Y axis represents the number of patients with dental implants. Blue represents the male patients and green represents the female patients. p value was found to be 0.27 (p>0.05) which is not statistically significant.

**Figure 5:**
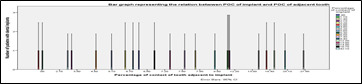
Bar graph representing the relationship between POC of implant and POC of adjacent tooth. The X axis represents the various POC of adjacent tooth to implant and the Y axis represents the number of patients with dental implants. p value was found to be 0.30 (p>0.05) which is not statistically significant.

## References

[2] Javed F (2013). Interv Med Appl Sci..

[3] Jambhekar S (2010). IJDA..

[4] Kim Y (2005). Clin Oral Implants Res..

[5] Cotrutca AM (2015). Rom J Morphol Embryol..

[6] Humphrey S (2006). Dent Clin North Am..

[7] Liu CW (2015). Biomed J ..

[8] Duraisamy R (2019). Implant Dent..

[9] Anbu RT (2019). Eur J Dent ..

[10] Sekar D (2019). Cancer Gene Ther..

[11] Sekar D (2019). Hypertens Res..

[12] Bai L (2019). Artif Cells Nanomed Biotechnol..

[13] Sivasamy R (2020). Vacuum..

[14] Sekar D (2020). Hypertens Res..

[15] Preethi KA (2021). Epigenomics..

[16] Preethi KA (2021). J Food Biochem..

[17] Bakshi HA (2019). Inflammation..

[18] Ezhilarasan D (2021). Drug Chem Toxicol..

[19] Thakur RS (2020). Environmental Toxicology..

[20] Ezhilarasan D (2019). Pharmacognosy Magazine..

[21] Samuel R (2019). Drug and Chemical Toxicology..

[22] Balusamy SR (2020). Biomed Res Int..

[23] Arvind PTR (2021). Orthod Craniofac Res..

[24] Venugopal A (2021). Semin Orthod..

[25] Ramadurai N (2019). Clin Oral Investig..

[26] Varghese SS (2019). J Dent Educ..

[27] Mathew MG (2020). Clinical Oral Investigations..

[28] Brunski JB (2000). Int J Oral Maxillofac Implants..

[29] Sequeros OG (2008). Journal of Oral Rehabilitation..

[30] Turp JC (2008). J Oral Rehabil..

[31] Ayuso-Montero R (2020). J Prosthodont..

